# Shifting Expectations of Novel Immunotherapy Treatments in Oncology: Practitioners' and Patients' Calibration Work in Conditions of Uncertainty

**DOI:** 10.1111/1467-9566.70076

**Published:** 2025-09-02

**Authors:** Julia Swallow

**Affiliations:** ^1^ School of Sociological Studies, Politics and International Relations University of Sheffield Sheffield UK

## Abstract

Immunotherapy cancer treatments stimulate individuals' immune systems to target and kill cancer, with the potential to extend survival time for individuals living with some forms of advanced cancer. Immunotherapies, however, generate uncertainties in relation to predicting prognosis and managing toxicities and the emergence of side effects during and post‐treatment. Drawing on interviews with practitioners and patients in an oncology clinic in the United Kingdom, this paper examines how these uncertainties, defined as epistemic and temporal, are articulated and negotiated in a wider context of shifting treatment expectations. Extending theorisation in the sociology of ‘low’ expectations, this paper demonstrates how practitioners and patients oscillate between high and low expectations of treatment to negotiate uncertainty. Patients are not passive consumers of hope and hype and do not always articulate high expectations of a pregiven and distant future, which requires recalibration in conditions of uncertainty. Instead, both practitioners and patients craft modest and personalised expectations and visions of the future, which at times involve anchoring to the present. Foregrounding both practitioners' and patients' accounts in theorising (re)calibration is important for understanding how expectations unfold and relate to uncertainties and with what consequences for the making of contemporary patienthood in the present.

## 1 Introduction

The writing is on the wall: cancer’s long and terrible reign as ‘The Emperor of All Maladies’ is soon coming to an end. The disease that kills more than eight million people worldwide has met a new foe capable of outsmarting and defeating it, and it’s been right under our noses the entire time: our own immune system.

(Canavan 2017)

This quote is the opening sentence from the book ‘A Cure Within: Scientists Unleashing the Immune System to Kill Cancer’, written by a US‐based immunologist, Neil Canavan. It encapsulates the ongoing scientific and clinical high hopes attached to these therapies, which, as they restore the function of the immune system to recognise and kill cancer, have the potential to extend survival time via long‐term cancer management or cure for patients with previously intractable or advanced cancers (see Canavan 2017). In the United Kingdom, two types of immunotherapy treatment are used either as standard of care treatment and/or are currently being tested as part of experimental clinical trials in the National Health Service (NHS). The first type is checkpoint inhibitors, also described as a type of monoclonal antibody or targeted treatment, to treat, for example, melanoma, urological and some types of lung cancer (see Cancer Research UK 2024). The second type is adoptive cell transfer therapies, including chimeric antigen receptor T (CAR T)‐cell therapy, which involves reprogramming the patient's own immune system T cells to be administered back into the patient to target their cancer (see Cancer Research UK 2024). The focus of this paper is on checkpoint inhibitors for advanced lung and urological cancers.

Checkpoint inhibitors stimulate the immune system to target cancer. In stimulating the immune system, however, they can cause side effects brought on by inflammatory and autoimmune complications if the immune system is *over*activated (Cancer Research UK 2023). Described as immune‐related adverse events (irAEs), these most frequently affect the skin, colon, endocrine organs, liver, joints and lungs, with more serious complications related to cardiac, neurological and renal impairments, and can occur long after treatment has stopped (Cancer Research UK 2023). Because these treatments enrol the immune system, uncertainties arise concerning the length of time that treatment works in the body (requiring constant monitoring), how to identify which patients will respond to treatment and how to predict when side effects may occur over time (see Langmuir et al. 2023). Tensions therefore arise between the wider hopeful discourse attached to immunotherapies, which emphasises progression towards cure, and a fixed future, and these (temporal and epistemic) uncertainties, which render patients' treatment futures with advanced cancer increasingly complex.

It is the purpose of this paper to explore how uncertainties are negotiated and entwined with patients' expectations of immunotherapy and the possibility of an extended future. As I show, immunotherapy generates epistemic and temporal uncertainties. I define epistemic uncertainty as related to the ways in which immunotherapy troubles practitioners' expertise and knowledge of cancer. I define temporal uncertainty as related to the ways in which immunotherapy disrupts the linearity of the cancer pathway (diagnosis–treatment–prognosis), where it has the potential to generate (future) side effects even when treatment has ended and extend cancer into the future indefinitely, making it difficult to predict treatment success over time. These two types of uncertainty are, of course, not mutually exclusive. Immunotherapy troubles practitioners' expertise, which makes it difficult for practitioners to provide certainty in the present (uncertainty extends into the future), and immunotherapy troubles the perceived linearity of the cancer pathway, which underpins practitioners' knowledge and decision‐making practices. Moreover, as treatment enrols a patient's own immune system, its effects are experienced differently from patient to patient, further impacting the difficulties in determining side effects and treatment success over time (see Tan et al. 2020).

Developing a wealth of literature that emphasises the relationship between the uncertainties and ambivalences characterising innovation in practice and expectations (Kerr et al. 2021; Gardner et al. 2015; Pickersgill 2011, 2019; Swallow 2017), I show how these epistemic and temporal uncertainties are negotiated through a dynamic interplay of high and low expectations. I argue that capturing how practitioners and patients oscillate between high and low expectations is important for extending theorisation of recalibration as involving more than practitioners enrolling patients in a regime of truth in conditions of uncertainty (Gardner et al. 2015). Instead, I extend theorisation in the sociology of (low) expectations in the context of understanding the exigencies of delivering novel therapies in oncology by decentring normative framings of the relationship between uncertainty and patients' (high) expectations of novel therapies that require recalibration in practice. I argue that in conditions of uncertainty, it is crucial to emphasise how practitioners and patients articulate high and low expectations in their accounts of immunotherapy *and* how patients calibrate modest and personalised expectations beyond positioning patients as passive consumers of hope and hype. As I show, expectations are therefore not fixed but shift over time in response to myriad uncertainty.

### 1.1 Uncertainties, Expectations and Cancer Medicine

Efforts to prevent, predict and extend (uncertain) futures drive contemporary biomedicine, particularly in oncology (see Baszanger 2012; Keating et al. 2016), enrolling patients in a biotechnical embrace and political economy of hope (DelVecchio‐Good et al. 1990). Physicians, researchers and corporate actors continually emphasise the promise of new or experimental medicines positioned as ‘silver bullets’ or ‘miracle cures’ (see Cortez and Halpin 2020), which reinforce a linear cancer pathway and progression towards long‐term survival, engendering the capitalist notion of a ‘disease‐free’ future (Clare 2017; Fortun 2008). The introduction of immunotherapies in oncology for intractable cancers with poor prognoses, and at the margins of progress in scientific research investment (e.g., lung and urological), encapsulates this hope. This political economy of hope, which drives biomedical efforts towards securing disease‐free futures, is not confined to the field of oncology but extends to other areas of degenerative disease research (see Clare 2017).

Literature within the sociology of expectations attends to the performative nature of such promissory visions and high expectations, or the ‘big’ futures of biomedical innovation projects, with particular emphasis on how these visions organise research development and investment, as well as clinical work (Michael 2017). Emphasis is placed on physicians, researchers and/or corporate actors in examining the promises and expectations, or future‐oriented discourses, attached to biomedicine. Increasingly, however, as Michael (2017) argues, there is a need to address the ‘little futures’ attached to innovation, including the lived experiences of individuals enrolled in the ‘biotechnical embrace’ of biomedicine (DelVecchio‐Good et al. 1990). The need to draw attention to the ‘little futures’ of innovation projects intersects with the wider sociological attention afforded to the sociology of low expectations, which accounts for the uncertainties and ambivalences characterising innovation in practice, alongside hope and hype (De Togni et al. 2023; Fitzgerald 2014; Gardner et al. 2015; Kerr et al. 2019; Pickersgill 2011; Swallow 2017; Tutton 2011). As Tutton (2011) argues, oscillating between promissory and less optimistic framings maintains momentum in biotechnology. More recently, in the context of AI and robotic technologies, De Togni et al. (2023) explore how professionals navigate and manage technoscientific expectations by demonstrating how ‘professionals articulate and navigate a range of high and low expectations and promissory and cautionary future visions’, which results in professionals constructing their own personalised visions of ‘acceptable futures’ (2009).

Central to work in the sociology of low expectations is theorising the tension between hyped visions of the future and the constraints and demands of delivering innovation in conditions of uncertainty. As a feature of biomedical and clinical practice (Fox 1980; Timmermans and Angell 2001), uncertainty has been theorised in relation to how it is navigated and managed by healthcare practitioners and patients in practice as a form of ‘uncertainty work’ (Moreira et al. 2009; Pickersgill 2011, 2019). Central to uncertainty work is the management of both epistemic and temporal uncertainties (see Pickersgill 2011, 2019; Cortez and Halpin 2020). Epistemic uncertainty has been theorised in relation to uncertainty concerning what clinical or biomedical knowledge ‘is’ in the context of, for example, neuroscience (see Pickersgill 2011, 2019). Temporal uncertainty has been theorised in relation to the way in which new trial drugs trouble the linearity of the disease pathway, particularly in cancer (Cortez and Halpin 2020). Scholars have also shown how uncertainty more broadly can be productive and enabling through generating particular kinds of action that serve to return the act of decision‐making to the clinic in the context of biomedical innovation (Latimer 2013; Reed et al. 2016; Swallow 2019) and/or through imagining alternative futures or social practices (Mackintosh and Armstrong 2020).

Analysing the relationship between expectations and uncertainty in clinical practice, Gardner et al. (2015) examine how expectations circulate as technologies are adopted in clinical practice in the NHS in the United Kingdom. Contributing to the sociology of low expectations, they show how patients' high expectations attached to innovation projects are ‘recalibrated’ by practitioners to account for uncertainty and the possibility of failure as well as ambivalence (Gardner et al. 2015). The authors emphasise how clinicians working at the ‘coal‐face of innovation’ engage in recalibration work with prospective patients (1001). As they note, ‘*recalibration* involves co‐constructing a vision of the future with each patient and encouraging the patient to adopt that vision and thus recalibrate their expectations with it’ (1001). In so doing, recalibration produces ‘modest, highly personalised, and uncertain futures’, and yet this ‘recalibration work’ is unacknowledged and unrecognised in the process of embedding translational medicine in practice (Gardner et al. 2015, 1001). This literature is underscored by what Moreira and Palladino (2005) describe as the regimes of ‘hope’ and ‘truth’ that accompany innovation projects: Hope performs the hype attached to innovation and truth accounts for the ‘realistic’ expectations of what these technologies do or do not achieve in practice.

In the context of cancer, Kerr et al. (2021) critically interrogate the promissory visions, or ‘high expectations’, of genomic medicine (tests, treatments and trials) as a new frontier in biomedicine while also foregrounding the on‐the‐ground expectations of those delivering genomic medicine in practice. As we show in our study of professionals engaged with genomic medicine, professionals negotiate the uncertainties of genomic research and the promise of precision in cancer as entwined with managing expectations in the clinic (Kerr et al. 2019). We describe ‘patient‐centred management of uncertainty to manage expectations and protect the integrity of their clinical judgement and expertise’ (234; see also Broom et al. 2009). In so doing, ‘lowering expectations (Gardner et al. 2015) and “tinging” their encounters with vulnerable patients with uncertainty (Moreira 2010) about the prospects of genomic research were part of their practices of care’ (Kerr et al. 2019, 236). Alongside foregrounding the on‐the‐ground expectations of professionals, we also focus on the lived experiences of patients and family members through elucidating the tensions and ambivalences at work in the making of genomic medicine (Kerr et al. 2021). Despite focusing on patients’ experiences of novel genomic technologies in cancer, the relationship between uncertainties and expectations across these wide‐ranging studies is primarily theorised in relation to the perspectives and negotiation work of practitioners and professionals.

In the context of delivering immunotherapy in practice, I build on this wealth of literature by centring both practitioners' and patients' accounts of crafting and negotiating expectations in conditions of epistemic and temporal uncertainty. In so doing, I extend theorisation on the relationship between uncertainty and expectations by showing how practitioners and patients construct and craft modest and personalised expectations that are not always tied to a pregiven future that frames wider miracle drug discourse. In the context of immunotherapy, through which epistemic and temporal uncertainties are generated, patients describe not always being guided by clinicians to recalibrate their high or unrealistic expectations (Gardner et al. 2015), nor do practitioners and patients describe ‘lowering expectations’ as part of practices of care (Kerr et al. 2019). Instead, recalibration involves more than practitioners enrolling patients in a regime of truth, as both practitioners and patients oscillate between high and low expectations of treatment in response to uncertainties that are generated by immunotherapy. At times, this involved anchoring to the present and bracketing out a fixed future. Rendering visible patients' high and low expectations serves to offer a more nuanced understanding of how patients' expectations unfold and relate to uncertainties, and with what consequences, as patients are enrolled in the making of contemporary patienthood in the present.

## 2 Methods

This article draws on semi‐structured qualitative interviews carried out with practitioners and patients across an oncology service in NHS Scotland as part of a Wellcome Trust fellowship (Grant No. 218145/Z/19/Z). This fellowship was funded as an ethnographic project involving observations of clinical practice and interactions between oncologists and patients; however, due to the COVID‐19 pandemic, interviews were the main source of data collection. I carried out 14 interviews with practitioners, including medical oncologists, consultant oncologists, clinical nurse specialists and research nurses. I also interviewed 16 patients across the lung and urology cancer service teams. This study was approved and granted NHS Research Ethics Committee approval (REC No. 20/NS/0062). Checkpoint inhibitors are prescribed as standard of care treatment for non‐small cell lung cancer and urological cancers, including renal cancer. All patients whose interviews I draw on for this paper were living with advanced cancer and were treated with checkpoint inhibitors when all other treatments had stopped working.

To recruit practitioners and patients in the lung and urology teams, I worked closely with a specialist nurse (lung and urology) and a consultant oncologist (lung) who acted as gatekeepers. To recruit practitioners, the gatekeepers introduced me to each clinical team, and from there I approached practitioners directly. I employed snowball sampling to gather a range of expertise and perspectives. To recruit patients, the specialist nurse approached suitable patients undergoing treatment in outpatient and inpatient clinics on my behalf, and I then followed up directly to provide further information about the project and to seek consent where appropriate. Pseudonyms are used throughout this article.

Interviews were semi‐structured, audio‐recorded and carried out between March 2021 and November 2023. Interviews lasted between 60 and 90 min and were conducted either online via Microsoft Teams or via telephone. I approached the interview schedules as guides, which allowed me to develop key points and enabled me to reflect on questions and refine where necessary. Interview audio files were transcribed verbatim. During interviews with practitioners, questions centred on the challenges and opportunities associated with novel immunotherapy treatments for clinical practice. I focused on the tension between the promise of these therapies for patients for whom all other treatments had stopped working and the uncertainties that were generated in practice. This included addressing how practitioners manage uncertainties around side effects and toxicities, with a specific emphasis on how checkpoint inhibitors differed from other therapies, such as chemotherapy and radiotherapy. During interviews with patients, questions centred on their views and perspectives on the role of the immune system in cancer and how they dealt with their treatment, including uncertainties associated with managing side effects, toxicities and prognosis. I adopted an inductive, reflexive approach to data analysis, developing themes and categories to illuminate the areas of inquiry of pertinence to the research. In line with feminist STS scholarship, I paid attention to the tensions provoked by the wider promissory and discursive framing of these novel therapies (Haraway 1988; Jain 2013; Murphy 2012; Puig de La Bellacasa 2011; Swallow 2024).

Across the following analysis, I draw on practitioners' and patients' perspectives and experiences of immunotherapy treatments to explore the impact of immunotherapy treatments on professional practice as well as patients' experiences of cancer treatment. In the first section, I set out how immunotherapy generates epistemic and temporal uncertainties that trouble existing clinical expertise and experience and which render practitioners' efforts to construct a future for patients increasingly difficult. I then go on to show how this uncertainty work (Pickersgill 2019) was conducted in response to the tension between the hopeful discourse around immunotherapy and the need to lower patients' (high) expectations of treatment to account for uncertainty. Practitioners, however, also mobilise uncertainty and pull patients into a regime of hope as they described the need to foreground the potential for treatment success. Oscillating between tainting expectations with uncertainty and pulling patients into a regime of hope captures only one aspect of the dynamic relationship between uncertainties and expectations. As I go on to show, patients, at times, described entering the clinic with low or no expectations of treatment, resisting or recalibrating both the high expectations tied to a wider miracle drug discourse (the success of immunotherapy) and, relatedly, recalibrating expectations of having a future when living with advanced and terminal cancer. Instead, as articulated in their accounts, patients crafted their own modest and personalised expectations of treatment, anchoring to the present to generate hope and provide provisional certainty while bracketing off a fixed future (see also Brown et al. 2015). I conclude by arguing that patients are not passive consumers of hope and hype where, alongside practitioners and in conditions of uncertainty, they described crafting modest and personalised expectations that are not necessarily tied to a fixed and pregiven future. In the context of delivering novel cancer therapies in practice, it is important to capture patients' accounts of recalibrating expectations and negotiating uncertainty in the present (see Kerr et al. 2021).

### 2.1 Negotiating Epistemic and Temporal Uncertainties

In this first section of the analysis, I draw out how immunotherapy, as it is delivered in practice, generates epistemic and temporal uncertainty. The practitioners I interviewed each emphasised how immunotherapy treatments, delivered for lung and urological cancers, challenge their expertise, cultivated over years of clinical experience. In particular, it troubles their understandings of, and existing ways of managing, cancer as related to toxicities, side effects and prognosis. This renders the future increasingly uncertain.

In order to contain uncertainty, practitioners each suggested that more time is needed to establish the necessary clinical experience to provide a level of certainty to patients with regard to side effects and prognosis. Although they recognised that there is always a degree of uncertainty with regard to new therapies (see Fox 1980), immunotherapy specifically disrupts their existing knowledge base. As one practitioner described, ‘*It's* *a completely new way of doing cancer treatment*’. Others described the importance of time for developing their skills and experience and for feeling confident in administering immunotherapy and navigating as well as resolving uncertainties. As one consultant oncologist remarked,

I’ve done 20 years of chemotherapy, and you know how these drugs work, and you know their side effects in. And you have a feel, it’s like cooking, you know what’s in your cupboard and you know how to use it. And then suddenly it’s almost like somebody put me in a different kitchen and asked me to cook.

### Consultant Oncologist 2

Similarly, as Consultant Oncologist 4 noted,

I guess that’s where clinical experience is probably quite important, when you give enough immunotherapy that you then have a feel.

Here, we see how practitioners' knowledge in administering cancer treatments and resolving unknowns and uncertainties is built up over time. As another consultant explained, uncertainty about the long‐term implications of these treatments can generate uncertainties and anxieties for patients who enter the clinic seeking a level of certainty about their treatment futures,

It’s very hard. Some patients psychologically, you know, they’re already trying to come to terms with having a cancer diagnosis where they don’t know what the future holds for them and then they come to you wanting answers and treatment and you’re giving them a treatment, you’re offering that but you’re saying to them, I can’t really tell you what this is going to do for you. We just have to wait and see. That can be quite tricky for patients.

### Consultant Oncologist 7

As Consultant Oncologist 7 explains, uncertainty is extended out into the future, which suspends cancer in time: ‘*we* *just have to wait and see*’. From the perspective of practitioners, adopting a ‘wait and see’ approach sits in tension with patients' efforts to seek clarity around the future in the present. Immunotherapy challenges practitioners' clinical expertise, and for practitioners, they described how this renders constructing a future for patients difficult, ‘*I can't really tell you what this is going to do for you*’, with the potential to generate further uncertainty for patients living with cancer. Across the interviews, (more) time was emphasised by practitioners as key to developing knowledge and was also mobilised as a means of containing uncertainty by pushing uncertainty into the future, effectively suspending cancer in time through orientation to the present.

These kinds of epistemic and temporal uncertainties have been discussed in relation to a wide range of new treatments and trials in cancer, particularly in genomic medicine (Kerr et al. 2019). What is distinctive about immunotherapy is that the long‐term effects of these treatments, as they enrol the immune system, are unknown and this renders efforts to construct a future for patients increasingly difficult. As Consultant Oncologist 7 explains,

Immunotherapy, anything can happen in the body and that’s what you have to try and explain to the patient and the fact that it can happen even after you’ve stopped the drug can be very difficult for the patient to understand.

As Consultant Oncologist 2 further iterates,

At the very best, these best experts are still a little bit in the dark, we don’t know from what these immunotherapy does in an adjuvant setting, whether we will live to regret it 20 years from now, when we record problems to you that we hadn’t foreseen, you know, that we can’t tell you because we just don’t have that expertise.

As I go on to show, this uncertain treatment landscape, which challenges practitioners' expertise, has implications for practitioners' roles as they describe the need, at times, to lower or recalibrate patients' (high) expectations. Although this echoes previous social science work in the sociology of low expectations, I also show that lowering or tainting expectations with uncertainty (Gardner et al. 2015; Kerr et al. 2019, 2021) captures only one aspect of the dynamic and shifting relationship between uncertainties and expectations when analysing how uncertainties generated by immunotherapy are negotiated and managed.

### 2.2 Shifting Expectations: High, Low and No Expectations

In the context of immunotherapy, uncertainties sit uncomfortably alongside the wider hope and hype around immunotherapy and the possibility of long‐term survival: a pregiven and fixed future. During interviews with practitioners, many emphasised how patients enter the clinic with high expectations of treatment. As Clinical Oncologist 6 remarked,

I think it’s often difficult, it raises people’s expectations, and I think we are talking about … well, I am talking about a lot of the media language is often miracle cures, transforming treatments, and it is very sort of headline‐driven … And yes, I think people hear that immunotherapy is this brilliant new treatment, they expect that they’ll respond, and they think they’re not going to have any side‐effects at all.

Echoed by Consultant Oncologist 9,

They will come [into the clinic] as something like, a cure has arrived, miracle drug, x, y, z. And then patients have … and to be honest, when patients come to clinic and know they will be talking about this, they may have maybe sometimes over‐expectations … And those type of news articles don’t mention about the toxicity, how many people need to stop the treatment, x, y, z.

As immunotherapy unfolds over time and both temporal and epistemic uncertainties and ambivalences also unfold, one practitioner also described having to recalibrate their own (high) expectations of treatment. As one research nurse remarked,

I think immunotherapy’s great. I think it’s probably less as amazing as I thought it was when I was first explained about it. I think there was definitely a – this is the miracle cure and we’re never going to have to give chemo again – and that’s definitely not happened.

### Research Nurse 2

As this research nurse explains, they lowered their own expectations of treatment as a ‘miracle drug’, likely to replace chemotherapy, in response to the exigencies of delivering these therapies in practice. Here, we begin to see how recalibration involves more than practitioners solely (re)shaping patients' expectations.

Alongside tainting expectations with uncertainty, practitioners also described mobilising uncertainty by drawing on the potential for treatment success to construct hopeful visions of the future and bring patients into the regime of hope. Consultant Oncologist 2 also described drawing on the potential for treatment success to generate hope when outcomes were unknown. Here, potentiality was a key feature of the need to contain uncertainty and generate hope,

There’s a whole set of things but I think sharing also brings hope and it’s trying to turn it from an uncertainty and an uncomfortableness to a positive, you know what, we don’t know and we’re trialling, hopefully, and you know what, I’ve got some patients doing amazing so what the heck, you know.

### Consultant Oncologist 2

Uncertainty was, at times, mobilised by practitioners as a means of providing or generating hope and constructing a provisional future for patients. This is demonstrative of a shift away from practitioners solely recalibrating patients' high or ‘over’ expectations of treatment to bring them into the regime of truth and instead reflects efforts to generate hope by cultivating potentiality and drawing on high(er) expectations. The regimes of both truth and hope are therefore not fixed but are themselves in the making as these novel treatments unfold over time.

Lowering or tainting expectations with uncertainty therefore captures only one aspect of the dynamic and shifting relationship between managing or negotiating uncertainties and the exigencies of delivering immunotherapy in practice. Practitioners remarked that patients who enter the clinic often have what they described as ‘no expectations’ of treatment related to the fact that they actively resist or are hesitant about the hopeful discourse of immunotherapy as a ‘miracle drug’. As Research Nurse 2 described,

They’re a bit hesitant of it rather than you know like ‘ohh it's amazing I'm getting this new drug’ sort of thing.

Echoed by Consultant Oncologist 8,

I don't get patients coming really with expectations greatly, I have to say.

As I go on to show in the final section of analysis, in conditions of uncertainty, patients do not passively consume hype or hopeful discourse around immunotherapy as a ‘miracle drug’ and instead articulate no and low expectations of treatment. As shown in their accounts, patients crafted their own modest and highly personalised expectations of treatment that were tainted by uncertainty and ambivalence. This form of (re)calibration enabled patients to anchor to the present and bracket out a (fixed) future (Brown et al. 2015). This does not, however, denote hopelessness but instead allows for a particular articulation of hope as patients construct their own modest visions of the future. Rendering visible patients' accounts of cultivating modest and personalised expectations of treatment, which at times involved orienting to the present, is crucial for understanding the impact of novel cancer therapies on patients' experiences of advanced cancer.

### 2.3 Patients' Calibrating Modest and Personalised Expectations

In a context of living with advanced cancer and where immunotherapy generates uncertainty around, as one patient described, ‘*whether the body will tolerate treatment*’, patients articulated their own hopes for immunotherapy that sit in tension with a ‘miracle drug’ discourse.

As Gill, who was diagnosed with bladder cancer, explains,

I just found out that … that it was new and it might work and it might not work. I didn’t approach it, ‘oh, this is a healing drug, this is my miracle in front of me’. No, if this works, gosh, I’m pleased.

David, who was living with kidney cancer, described how his expectations for treatment success were similarly modest,

No, I didn't change that. The cancer is there. That is a fact. You can't change that. But the way … I didn't expect the immune … I didn't expect immunotherapy to completely eradicate the cancer. All I wanted was that … my … you know, as I said earlier, a major win for me was stopping a progress. But … so you’re not looking for a miracle, you know.

Here, we see how Gill and David, both living with advanced cancer, construct their own modest or realistic as well as personalised expectations of immunotherapy and treatment success. Situated within a wider context of epistemic and temporal uncertainty concerning whether the body will ‘*tolerate treatment*’, as one patient explained, and for how long, patients articulated modest as well as personalised expectations of treatment ‘success’. As David iterates, ‘*a* *major win for me was stopping a progress*’.

As I will show, these muted, modest as well as personalised expectations enabled patients to anchor to the present with an emphasis on living day to day with cancer, bracketing off a fixed and pregiven future (see also Brown et al. 2015). Here, bracketing off expectations of having a future effectively suspends cancer in time. During an interview with Jack, who had recently completed a course of immunotherapy for lung cancer, he spoke about the difficulty anticipating when or if treatment will stop working in the body, extending uncertainty out into the future.

The trouble with immunotherapy is, well, you don’t know how long it’s going to last for, nobody seems to know, you know, but then, you know, you could get run over by a bus tomorrow, you know, so every month is a bonus, isn’t it, I suppose?

As Jack explains, mediating epistemic and temporal uncertainty is achieved through anchoring to the present and also bringing into being a future in a context where time is limited, ‘*every* *month is a bonus*’. Others spoke of actively bracketing off the future, aware that to look too far into the future could generate ‘*false hope*’, as Gill further explained,

I’m not a pessimist by nature, but I’m not looking too far to the future because that again to me is false hope.

Well, I don’t believe in looking too far into the future because anything can happen.

For Rachel, who was living with renal cancer and had been enrolled in an immunotherapy clinical trial, this involved not focusing on time limits and anchoring to the present,

We don’t really talk much about prognosis now. It’s not something that’s of overwhelming importance to me because I’m trying to take things day by day and try to make good memories and just not be paranoid about time limits.

This orientation to the present related to the crafting of modest or realistic expectations attached to having a future could be experienced as liminality (Jain 2013). Yet, here, in order to account for uncertainty, the present emerged as a meaningful temporal space through which to craft modest expectations and bracket off a pregiven and fixed future. In anchoring to the present and crafting modest expectations, for a number of patients I interviewed, immunotherapy also had the potential to generate a future that previously did not exist, and this meant bringing patients back into the regime of hope and *raising* expectations. For a number of patients whose cancer had progressed, immunotherapy meant that it was no longer detectable, where, in effect, long‐term control was being realised in practice. This had the potential to render the future increasingly uncertain, as prognosis became more difficult to predict, but conversely, it also meant that patients were faced with raising their (low) and modest expectations of having a future and to pull themselves (back) into the regime of hope.

As Mark, who had initially been given 3 weeks to live prior to starting immunotherapy for advanced lung cancer, explained,

And we did ask him ‘how long have I got?’ And he’s now saying he doesn’t actually know because he … ‘cause we’d asked him to be blunt about it and he was blunt, he says that he doesn’t actually know, he says, because he wasn’t expecting to see me now’.

Similarly, Adrian, who was diagnosed with lung cancer and was being treated with immunotherapy, remarked,

It was, I mean, to be honest with you, I mean, it was beyond all expectations. It was, it was just beyond all expectations, it just beyond all expectations … to say that it’s got the cancer under control.

In this final section, I have begun to show the ways in which patients described crafting their own modest, ‘realistic’ and personalised expectations of treatment success and of having a future in response to the epistemic and temporal uncertainties that are generated by immunotherapy. To manage these uncertainties, patients, at times, described resisting the wider ‘miracle drug’ discourse (recalibrating expectations of treatment success) and bracketed out a fixed and pregiven future (recalibrating expectations of having a future). In so doing, anchoring or reorienting to the present—extending out uncertainty into the future while also emphasising the importance of living day to day with advanced cancer. Alongside this calibration work and crafting modest, realistic as well as personalised expectations, patients, at times, also articulated beyond expectations, which meant pulling back into a regime of hope if treatment was successful. Expectations are therefore contingent and shift over time as treatment unfolds over time in conditions of uncertainty. Situating both practitioners' and patients' accounts in theorising (re)calibration serves to offer a more nuanced understanding of the relationship between uncertainties and expectations when analysing the exigencies of delivering novel therapies in practice, particularly those that trouble the linearity of the cancer pathway and reshape co‐constructed visions of a fixed future.

## 3 Discussion

In the context of immunotherapy, expectations, which are both discursive and deeply material practices, shift over time as treatment unfolds over time, shaping how cancer is managed and experienced and how visions of the future are (and are not) constructed within wider conditions of uncertainty. Drawing on the case of immunotherapy, I have extended theorisation concerning the relationship between uncertainties and expectations through emphasising the contingency of expectations as they shift over time and by including the accounts and perspectives of practitioners and patients in theorising (re)calibration. Gardner et al.'s (2015) work is crucial for rendering visible the unacknowledged work of recalibration in managing patients' expectations as novel therapies are delivered in practice. This paper further emphasises the need to pay attention to patients' expectations in conditions of uncertainty but has centred patients' articulations of high and low expectations and resultant (re)calibration work in the analysis. This is not as passive consumers of hope and hype but as key actors in negotiating emerging epistemic and temporal uncertainties through (re)calibration work, which involved articulations of the need to craft modest, realistic as well as personalised expectations. Patients described working to negotiate uncertainty through lowering their own expectations and/or raising expectations should immunotherapy extend futures. At the same time, therefore, we see how the regimes of truth and hope and high and low expectations are not fixed but are (re)calibrated by practitioners *and* patients in a wider context of uncertainty. Paying analytical attention to patients as key actors in calibrating expectations is also a reminder for us as social science scholars to decentre biomedical and normative framings of hope and hype which have the potential to be reified in a wider context of conducting research in biomedical spaces.

Across the analysis I demonstrated how immunotherapy generates what I describe as epistemic and temporal uncertainties. Immunotherapy troubles existing clinical expertise and experience, which renders the future increasingly uncertain as side effects occur over time and treatment works in the body over time, making it (further) difficult to predict prognosis. Practitioners and patients described how these entwined epistemic and temporal uncertainties render the future increasingly uncertain, effectively suspending cancer in time. As I showed, these uncertainties sit in tension with (some) patients' high hopes and expectations, which feed into the clinic. On the one hand, high hopes and expectations for treatment success feed into the clinic requiring ‘recalibration’ through tainting or lowering patients' seemingly fixed expectations (see Gardner et al. 2015). On the other hand, and this is key to extending literature in the sociology of low expectations, both practitioners and patients articulate low as well as no expectations of treatment to manage uncertainty. Patients, at times, actively resist a wider miracle drug discourse and craft their own modest as well as personalised expectations of both treatment success and of having a future through anchoring to the present and bracketing out a fixed future to provide provisional certainty. Moreover, patients articulate what they describe as beyond expectations when treatment is successful—here, uncertainty could also be productive in that it has the potential to generate hope if certainty of not having a future is temporarily lifted. As articulated in their accounts, practitioners and patients therefore move continuously between the regimes of truth and hope. As I show, in response to myriad uncertainty, expectations are not fixed but shift over time.

Theorising the tension between hype and hope and the demands of delivering these treatments in practice is therefore not solely concerned with practitioners constructing visions of the future for patients that are either tainted with uncertainty or that perform hope and hype. Instead, theorising this tension requires acknowledgement of how patients articulate and craft modest, realistic and personalised expectations that emphasise both the present as well as a hoped‐for future. Patients living with advanced cancer, at times, enter the clinic with low or no expectations of treatment, which enacts experiences of cancer that are oriented both towards crafting modest futures and present care. Moreover, as immunotherapy enrols a patient's own body in treatment as a targeted or personalised therapy, it is important to consider how the concept of personalisation extends out to navigating or co‐constructing expectations and visions of a particular personalised future. Here, expectations are highly contingent and situated, and calibration requires enacting new forms of patienthood in conditions of uncertainty. Acknowledging how expectations shift over time has important implications for further understanding and theorising the making of contemporary patienthood as novel therapies are delivered in practice.

Although this paper has situated practitioners' and patients' accounts of immunotherapy as key to theorising (re)calibration, further research could explore how the regimes of truth and hope and high and low expectations are co‐constructed, mutually shaped and (re)calibrated by practitioners *and* patients in clinical practice with an emphasis on doctor–patient interactions.

## Author Contributions

**Julia Swallow:** conceptualization (lead), formal analysis (lead), funding acquisition (lead), investigation (lead), methodology (lead), project administration (lead), writing – original draft (lead), writing – review and editing (lead).

## Consent

All patients consented to be interviewed as part of the study.

## Conflicts of Interest

The author declares no conflicts of interest.

## Data Availability

The data that support the findings of this study are openly available in Edinburgh DataShare at https://journals.sagepub.com/doi/epub/10.1177/03063127231199217.
